# “My feet are no longer swollen”: Testing a co-designed podoconiosis protective footwear among rural female farmers in Rwanda

**DOI:** 10.1371/journal.pgph.0007114

**Published:** 2026-10-06

**Authors:** Gloria Igihozo, Leila Dusabe, Jeanne Uwizeyimana, Jamie Boiles, Tonya Huston, Janna M. Schurer

**Affiliations:** 1 Center for One Health, University of Global Health Equity, Butaro, Rwanda; 2 Department of International Health and Sustainable Development, Celia Scott Weatherhead School of Public Health and Tropical Medicine at Tulane University, New Orleans, Louisiana, United States of America; 3 Heart and Sole Africa, Musanze, Rwanda; 4 DuHope, Kigali, Rwanda; PLOS: Public Library of Science, UNITED STATES OF AMERICA

## Abstract

Podoconiosis is a disabling neglected tropical disease (NTD) caused by prolonged occupational exposure to alkaline red clay soil, disproportionately affecting female farmers in Rwanda. Although preventable through the consistent use of protective footwear, such as farming boots, high costs and limited availability at local markets impede rural farmers’ access to appropriate footwear. Among female farmers, gender norms and cultural expectations further constrain their ability to access and wear farming boots. This qualitative community-based participatory project aimed to test a women-centered farming boot, co-designed by participants and the research team, to address previously identified barriers to protective footwear access among rural Rwandan farmers. Boots were distributed for testing, to male and female farmers, in four villages with the highest podoconiosis prevalence across Rwanda. This was paired with education campaigns and other informational materials. Sixteen audio-recorded focus group discussions were subsequently conducted to explore farmers’ experiences with the boots. Farmers reported that receiving the footwear encouraged a more consistent use of farming boots, with female participants expressing feelings of pride and an emerging sense of empowerment associated with owning and wearing the boots. While community reactions to the boots varied, most female farmers noted that restrictive gender norms and expectations related to boot wearing persist but do not deter them from wearing the boots. Findings reiterate the importance of gender-sensitive, culturally tailored, and locally driven solutions in preventing podoconiosis and other skin NTDs. Future podoconiosis eradication strategies should strive to prioritize equitable access to protective footwear and address underlying social norms that limit women’s access to preventive tools.

## Introduction

Podoconiosis is a non-infectious neglected tropical disease (NTD) that is characterized by bilateral swelling of lower limbs, lesions, and nodular growths [1,2]. It is likely caused by long-term exposure to irritant particles in red clay soil that penetrate the skin and induce inflammation, leading to chronic non-filarial lymphoedema [2]. The disease disproportionately affects subsistence farmers in low-income regions of Africa, Central and South America, and Southeast Asia who farm barefoot and have limited access to water [3,4]. Beyond its physical manifestations, podoconiosis is a disabling disease that is associated with stigma, reduced economic productivity, psychological distress, and social exclusion, contributing to persistent cycles of poverty for affected households [5–7]. As such, effective prevention and management strategies are essential for reducing disease burden, preventing disability, improving quality of life, and mitigating the social and economic impacts of podoconiosis.

In sub-Saharan Africa, podoconiosis remains endemic across 12 countries, with the largest prevalences reported in Cameroon (8.1%), Ethiopia (7.5%), Uganda (4.5%), and Kenya (3.9%) [8,9]. Rwanda reports a national prevalence of 68.5 cases per 100,000 individuals, with women twice as likely to be affected as men [8,10,11]. In Rwanda, knowledge of podoconiosis etiology and management is often low among healthcare providers, impacting definitive diagnoses and patient care [12]. In such settings, prevention strategies that reduce exposure to irritant soil particles, including the promotion of protective footwear, remain critical components of podoconiosis control efforts. These interventions align with the World Health Organization (WHO) 2021 – 2030 NTD roadmap, which emphasizes global and national efforts that prioritize innovative preventive, community-centered, and scalable interventions that extend beyond the healthcare sector [13]. By preventing disease onset and progression, footwear-focused interventions may complement broader efforts to integrate podoconiosis prevention and management into routine community and primary healthcare services.

Podoconiosis is preventable through consistent wearing of protective footwear and regular maintenance of foot hygiene [5]. In Europe and North Africa, behavior change communication and increased adoption of shoe-wearing contributed to the elimination of the disease [14]. Shoes and socks provide a protective barrier for the skin, reducing contact with irritant soil particles. To be effective, footwear must cover both the soles and top of the feet, and they must also provide a sufficient barrier in scenarios involving manual labor that increases exposure to soil, such as farming [1]. Examples include closed leather shoes and plastic or rubber boots. In Ethiopia, interventions aiming to reduce disease onset and improve case management have focused on shoe distribution among at-risk communities [15–18]. For instance, Mossy Foot International has set up a footwear workshop that manufactures and distributes over 400 pairs of shoes monthly, which has increased shoe-wearing behaviors and improved case management within these communities [15,16]. Although footwear distribution initiatives have demonstrated promise in endemic settings, national podoconiosis prevention strategies in Rwanda primarily emphasize community education on the benefits of wearing shoes [19].

Despite evidence of the effectiveness of protective footwear in podoconiosis elimination, socioeconomic and cultural barriers limit the success of prevention programs in Rwanda and sub-Saharan Africa [5]. Protective footwear available at local markets can be expensive and unaffordable to high-risk populations, contributing to the continued use of sandals and other open shoes [20,21]. Women in rural Rwanda and Ethiopia have also reported limited access to protective footwear, due to a lack of suitable options at village markets and local stores [20–22]. Further, consistent shoe-wearing is not widely practiced in rural Rwanda, especially among women who are discouraged by a complex interplay of sociocultural and gender-based norms, power dynamics, and community backlash [11,21]. Women’s limited control over decision making and household finances are among the major factors hindering rural female farmers from purchasing and wearing boots [21]. These findings suggest that increasing awareness alone may be insufficient to effectively control podoconiosis, further highlighting the need for interventions that consider and respond to the needs and contextual realities of at-risk communities.

Achieving the control and elimination of podoconiosis requires multidisciplinary innovations that protect the skin and minimize exposure to irritant soil [23]. In endemic communities, gender transformative approaches can help address barriers that limit rural women’s access to protective farming footwear. One such approach would be the use of Target Product Profiles (TPPs), which can guide the development of products that respond to the needs and preferences of populations at risk of podoconiosis. TPPs outline the preferred specifications of a product, prior to development, according to its target population [24]. Incorporating the TPP approach into NTD programs in Rwanda could facilitate the development of products that address gender disparities and meet the health and practical needs of farmers. Previously, we identified barriers to protective footwear access and described the specific footwear preferences of rural farmers and individuals living with podoconiosis in Rwanda [21]. However, little is known about users’ experiences with footwear prototypes developed from these experiences or their acceptability in farming settings. Guided by the principles of gender transformative approaches, this community-based participatory research project, thus, aimed to (1) test a culturally and ergonomically acceptable footwear prototype that was designed following the previously identified footwear preferences and (2) characterize rural female farmers’ experiences wearing the designed footwear prototype.

## Methodology

### Ethics statement

Approval to conduct this research was granted by the University of Global Health Equity’s Institutional Review Board (Ref: UGHE-IRB/2021/055) and the Rwanda Ministry of Health’s National Health Research Committee (Ref: NHRC/2022/PROT/016). Further approval to conduct the study was sought from individual district offices before starting data collection. All participants provided informed written consent prior to participation.

### Setting

Rwanda is a densely populated country in East Africa with 449 inhabitants per square kilometer and a total population of over 13.2 million residents [25]. The country’s economy is largely agrarian; the agricultural sector employs 72% of the working population and more than 70% of the total population are subsistence farmers [26]. Overall, the agricultural sector contributes 33% to Rwanda’s national GDP, with tea, coffee, plantains, potatoes, beans, and cassava being the most widely cultivated crops. Although women account for 79% of the agricultural labor force, reports have indicated that they are less productive compared to their male counterparts [27]. Women’s farming productivity is constrained by limited access to agricultural inputs such as technology, as well as household power imbalances and discriminatory social norms that limit their decision-making power and control over land and income [27].

Rwanda is divided into four administrative provinces (Northern, Southern, Eastern, Western) and the City of Kigali. The provinces are further subdivided into hierarchical administrative units: district, sector, cell, and village. Districts with the highest podoconiosis prevalence in each province, according to the most recent national mapping survey [11], are Nyamasheke (Western Province), Musanze (Northern Province), Nyagatare (Eastern Province), and Nyaruguru (Southern Province) (Fig 1).

**Fig 1:**
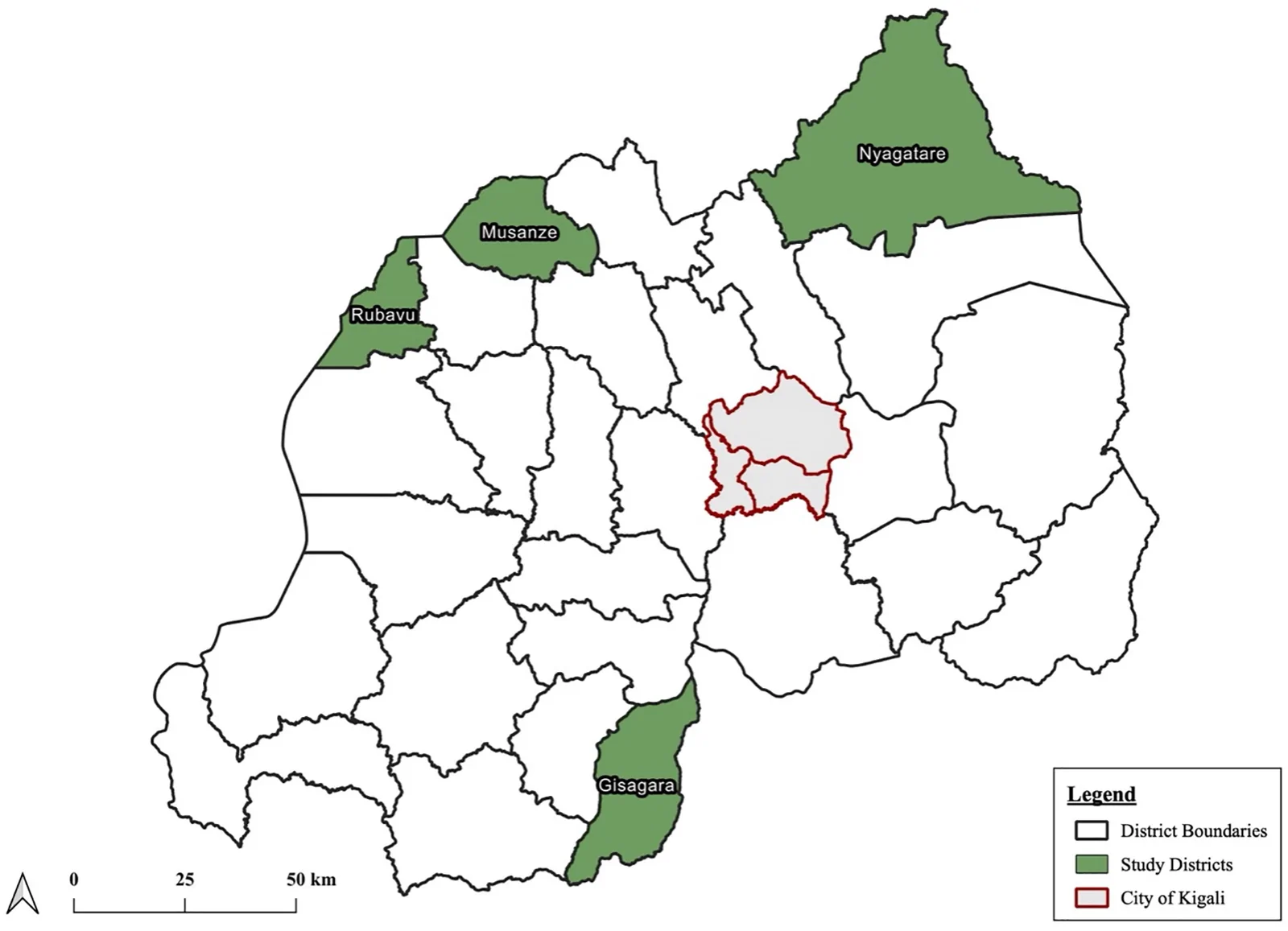
Map of Rwanda, highlighting study districts and the City of Kigali. Administrative boundaries dataset obtained from the “Humanitarian Data Exchange (HDX), https://data.humdata.org/, CC BY 3.0 IGO.” The map was generated and modified by the authors for this study.

### Partnership

This project was a collaborative effort between the University of Global Health Equity (UGHE), Heart and Sole Africa (HASA), and DuHope. HASA is a community-based non-governmental organization (NGO) that provides podoconiosis care and management in Rwanda. DuHope is a Community Benefit Company (CBC) whose aim is to provide opportunities for Rwandan women to leave sex work by equipping them with job skills and providing employment. The UGHE team designed the study and took lead in the implementation and coordination of all activities. The team at HASA advised on the development of data collection tools, assisted with data collection, and led community-based education campaigns. The team at DuHope designed the prototypes and led the manufacturing process for the final boot to be tested by study participants.

### Prototype development and testing

A women-centered farming boot was designed in partnership with DuHope, using farmers’ footwear preferences from our previous assessment [21]. The organization conducted an analysis of available raw materials in Rwanda and developed the first boot prototypes. Six prototypes were developed and went through a series of testing and modifications by DuHope staff, before a final prototype was approved for distribution to study participants. The prototype design process used an iterative approach, where prototypes were presented to all partners to obtain expert opinions on their alignment with participants’ preferences and suitability for minimizing direct contact with soil. The final prototype aimed at balancing functionality, design, and cost. After the study team had approved the final prototype, it was sent to a local manufacturer for production.

Boots were distributed to 148 rural farmers (110 women and 38 men) living in four cells with the highest podoconiosis prevalence in the four aforementioned districts, to be tested for 1 month. Since previous qualitative assessments revealed that women had less access to boots, providing them with boots offered opportunities to challenge gender norms and address identified barriers to wearing protective footwear. Participants were instructed to wear the prototype daily during their regular activities (i.e., farming, household chores, market runs, and more) and make notes of their observations of the boot and how they felt. During the boot distribution, HASA provided community education on podoconiosis that included its causes, management, prevention, and addressing gender norms and myths associated with boots. Participants also received empowerment materials: bracelets inscribed with the slogan “Ibirenge Bizima” (Fit Feet) and a Kinyarwanda language comic book narrating a female protagonist’s journey of learning about a mysterious disease that caused her friend’s feet to swell (Fig 2). The comic book was used solely as an educational aid, to support community engagement and facilitate discussions on podoconiosis prevention and footwear use; evaluation of the comic book itself is beyond the scope of this study. After the trial period, the study team returned to the communities to evaluate the prototype and understand participants’ experiences wearing the boots.

**Fig 2:**
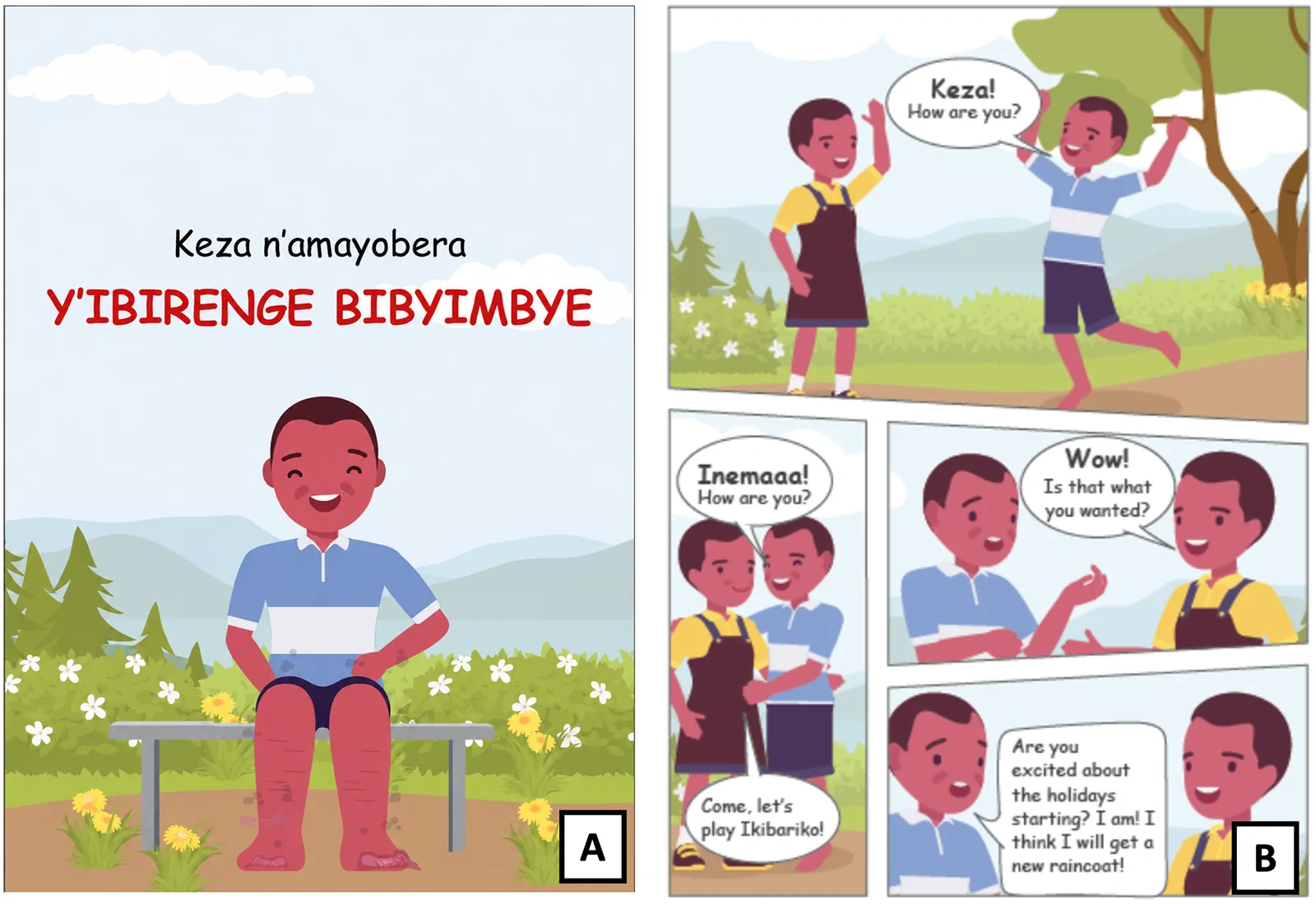
Comic book developed by the research team for community education campaigns; A- Kinyarwanda version, B- English translation.

### Study design and sampling

A qualitative, community-based participatory action research utilizing focus group discussions (FGDs) was conducted between June and July 2023. The study was conducted in the cell with the highest podoconiosis prevalence per province. These cells were identified in the first phase of this study [21], through the Rwanda Biomedical Center’s database of podoconiosis cases across Rwanda [11]. Participants had been purposively recruited between 14/04/2022 and 24/06/2022, through the help of community health workers, who provided a list of all residents in their respective villages. Potential participants were screened for eligibility and approached by the research team. Individuals were eligible if they were low-income (belonged to Ubudehe categories 1 and 2) farmers aged between 18 and 65 years, had resided in the study area for at least one year, and reported crop and animal production as their primary source of income. Individuals who were affected by medical conditions that prevent them from wearing commercially available footwear, such as individuals with late stage (Stage 4) podoconiosis were not included in the sample. A total of 4 FGDs were conducted per cell (Table 1), with each FGD having 8–10 participants as per standard guidelines [28]. In total, 16 FGDs were conducted with 148 farmers across the four study sites.

**Table 1:** Focus group discussion participant composition at each study site [21].

FGD #	Gender	Age range (years)	Health status
1	Female	18-30	Healthy
2	Female	31-65	Healthy
3	Female	18-65	Stage 1 podoconiosis^1^
4	Male	18-65	Healthy

^1^Characterized by mild swelling that is reversible overnight or with leg elevation.

### Data collection

A semi-structured FGD discussion guide with 7 questions was developed to assess male and female farmers’ experiences wearing the farming boot (S1 Text). The tool provided guided reflections on the features of the footwear that participants liked or disliked, potential improvements that could be made to the boot, and how suitable the shoes are for various activities. Discussions also explored how wearing the boots changed participants’ perspectives on who should wear farming boots, community reactions to women who wore the boots, and any observed changes in individual shoe-wearing. The FGD guide was developed in English and translated to Kinyarwanda by the UGHE and HASA teams. The tool was pre-tested with two groups of female and male farmers outside the study area to assess question clarity, with adjustments made based on their feedback.

Audio-recorded FGDs were conducted in Kinyarwanda by two members of the HASA team (J.U. & E.N.), in a comfortable and private space that would encourage participants to share their experiences. Each discussion lasted 1–2 hours. One team member facilitated the session while the other one took notes. These roles were alternated from one FGD to another, to obtain richer insights [28]. Field notes were taken to capture key discussion points and non-verbal cues.

### Analysis

Audio recordings were transcribed verbatim and translated to English by a team of three data collectors. Independent open reading of four FGD transcripts was done by two members of the UGHE team (G.I. & L.D.) to develop a draft codebook. The team convened to compare codes and develop a final codebook for analysis. To ensure consistency in the application of the codes, six transcripts were independently coded before meeting to compare codes, discuss challenges, and refine the codebook. To preserve the richness and authenticity of the original data, particularly for concepts related to gender dynamics and cultural norms related to boot-wearing, the research team coded the transcripts in Kinyarwanda and translated only the quotes selected for inclusion in the manuscript. All transcripts were coded independently, after which the researchers met to compare codes, identify themes, and discuss any discrepancies in code application or interpretation. Discrepancies were resolved through consensus and the coding framework refined iteratively, throughout the analysis process. Coding was done inductively and iteratively using Dedoose software (Version 9.2.5), following the constant comparative method [29]. To ensure accurate representation of findings, representative excerpts were translated by G.I., being careful to maintain their original meaning. Where applicable, the study methods and reporting have been completed in accordance with the Standards for Reporting Qualitative Research (SRQR) checklist [30].

### Rigor and reflexivity

To enhance the trustworthiness of the study and its findings, several strategies were used. Credibility was strengthened through prolonged engagement with farmers- introductions to build rapport, community education, boot distribution sessions, and endline assessments- as well as researcher triangulation. Additionally, credibility of findings was maintained through independent coding of transcripts by multiple coders, regular discussions to compare codes and themes, and providing thick descriptions of the research context and participants’ experiences. Dependability was supported through a detailed description of the methodology and writing reflective memos throughout the analytic process to document analytic decisions and emerging interpretations. Confirmability was enhanced by maintaining detailed records of coding decisions, as well as resolving discrepancies through discussions to reach consensus.

The research team included investigators with expertise in public health, qualitative research, gender, and neglected tropical diseases. Four members of the research team (G.I., L.D., J.U., E.N.) were fluent Kinyarwanda speakers and familiar with the local and cultural context, facilitating data collection, coding, and interpretations. Throughout data collection and analysis, the team engaged in regular discussions to evaluate how their respective backgrounds, experiences, and assumptions could influence their approach to the research, to ensure that findings remained grounded in participants’ perspectives and lived experiences.

## Results

### Designed footwear prototype

The protective footwear prototype co-designed with rural farmers was a waterproof rubber boot in three colors: green, black, and blue. The boots were mid-calf in height, had grooved soles, and a soft padded inner sock that could be washed and dried within an hour. The top of the boot was open and had laces, to enable people to adjust the boot to their preference. To enhance their aesthetic appeal, the top edge of the boot was lined with kitenge fabric to match the traditional and daily attires of rural women. The kitenge was covered with a transparent plastic material to ease cleaning and enhance durability (Fig 3).

**Fig 3:**
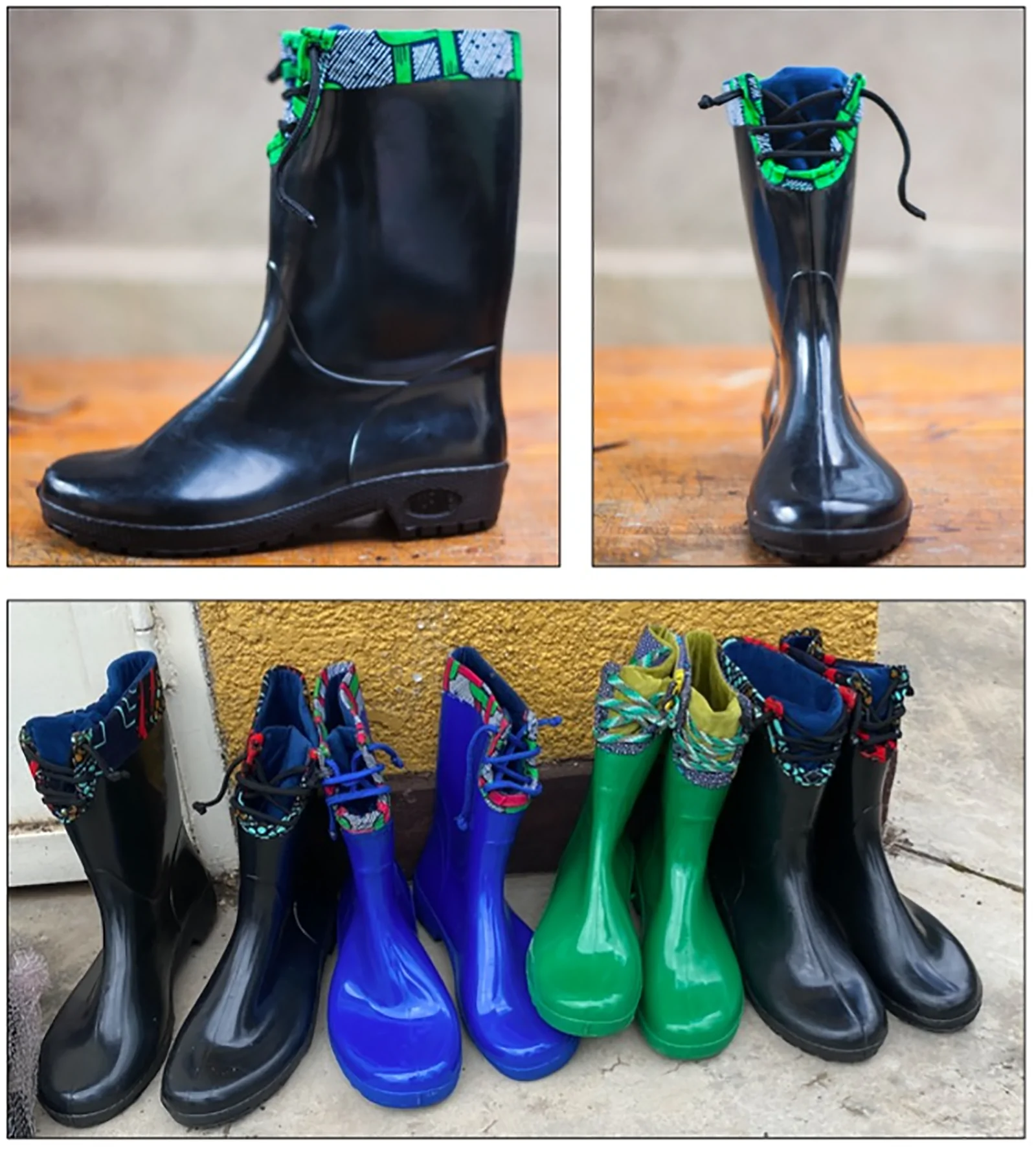
Farming boot prototype, co-developed by the research team and farmers. Photo credit: J.B..

### Themes

Across the four study cells, all farmers that took part in the first phase of the project participated in the prototype testing (110 women and 38 men). From the interviews, four themes were identified as central to understanding participants’ experiences with the farming boots:

1. Behaviors and perspectives related to shoe-wearing changed.
2. Gender-based norms and stereotypes related to boot wearing persist but do not deter female farmers from wearing the farming boots.
3. Female farmers felt happy, empowered, and proud to wear farming boots.
4. Some farmers who wore the farming boots were labeled as diseased by their communities.

#### Theme 1: Behaviors and perspectives related to shoe-wearing changed.

##### Perceived improvement in feet health.

Participants reported that receiving farming boots and engaging in the education campaign encouraged more consistent shoe-wearing and improved their perceptions of healthy feet. Having access to boots gave them extra footwear which could be dedicated to farming activities and household chores. Participants living with podoconiosis perceived that regularly wearing boots was associated with improvements in their symptoms, with many reporting reduced swelling and itching, and an improved ability to wear normal shoes.

*“I have always had problems with podoconiosis. But ever since receiving these shoes and wearing them, I have noticed the swelling going down. The other day, I went to the clinic, and they exclaimed, ‘ah, your podoconiosis has been cured!’ And I replied that it’s because of these boots. Boots are great, my feet are no longer swollen, they are gradually getting better.” (Female, 18-65 years, Nyagatare)*

*“The fascinating thing about these shoes… I used to have a hard time affording closed shoes such as boots that I could wear during chores and would work barefoot. Then I would have issues with cracked heels, athlete’s foot, and my feet would swell. But ever since receiving and wearing these boots, things are getting better. I have fewer issues.” (Male, 18-65 years, Nyagatare)*

##### Changes in perceptions about women who wear boots.

Female participants, especially, mentioned that understanding the boots’ health benefits contributed to a mindset change and made them feel more comfortable wearing farming boots. Male and female respondents testified that this change in mindset extended to their families and communities, which inspired other women to buy and wear boots without any fear of being judged.

*“There have been some changes [in mindsets]! We women, we used to think that boots are only for men. But this has changed, women are now included.” (Female, 18-30 years, Nyagatare)*

*“I used to see someone who wears boots as someone superior or different from others. But you taught us that wearing boots is protecting ourselves against infections and diseases, and that it is for everyone. After receiving them and starting to wear them, we also felt safe and protected and we were not ashamed. In addition to that, women’s mindsets have improved, we see [boots] as any other types of shoes.” (Female, 18-30 years, Musanze)*

*“Giving these boots to the women in my cell encouraged other women to also buy boots. They now go in the farms wearing them, without any kind of shame or embarrassment.” (Male, 18-65 years, Rubavu)*

##### Comfort and usability of the farming boots.

Respondents perceived that the boots were comfortable, provided protection against physical injury, promoted hygiene, and could be worn during different activities such as farming, rearing livestock, fetching water, and going to the market. Altogether, these factors encouraged boot wearing. However, a few participants stressed that boots are only ideal for farming and cannot be worn during social activities, such as weddings, church, or attending the market, because of the types of outfits worn during these events.

*“I have never had any issues with these boots; in fact, I have worn them in so many activities, like when harvesting hay for the cattle. Oh God, I wear them all the time and now, I no longer have the issues that I used to have … Maize stalks used to hurt my feet, I would step on stones and get cuts on my toes, thorns would pierce me… listen, we no longer encounter sticks and broken glass. These shoes have provided me with protection.” (Female, 31-65 years, Gisagara)*

*“I find it uncomfortable to wear boots to the market … There are times you go to the market wearing iribaya [a traditional kitenge dress], so wearing boots with it would not be a good match … you see, when you are rearing livestock or farming, there are specific clothes you wear. So, even at the market, there are clothes you wear that require you to wear different shoes.” (Female, 31-65 years, Nyagatare)*

##### Changes in perceptions related to risks of damaging crops.

Previously, farmers identified a fear of damaging crops as a key reason for not wearing boots. After testing the farming boots, participants acknowledged that their personal experiences did not support this perception. Nonetheless, many farmers emphasized that they had to be cautious and exercise care while farming. A few participants still worried about damaging crops and were fearful of wearing boots during some farming activities, such as harvesting. Consequently, some of the respondents mentioned that they would remove the shoes and walk in the farms barefoot.

*“For years, people used to say that these boots would damage crops. But from our experience, that is not true!” (Female, 18-65 years, Rubavu)*

*“To harvest [some] fruits or seeds, you need to remove the boots so that they don’t damage them. But in some other farming activities, this is not an issue. Personally, I have never removed these boots, unless I was harvesting potatoes to take to the storage area. That’s when I remove them, so that I don’t destroy the potatoes.” (Male, 18-65 years, Musanze)*

#### Theme 2: Stereotypes and gender-based norms related to boot wearing persist but do not deter female farmers from wearing the farming boots.

Farmers mentioned that stereotypes and norms related to who should wear boots and what is appropriate for women to wear persist. For female participants, this was manifested through criticism of the boots, being labeled as disrespectful, being confused with agronomists, and misconceptions that they are rich simply because they owned boots. One woman also said that having the boots has reduced her employment prospects, by making it harder to get casual farming jobs.

*“When I wear them and I go in the community, people confuse me for a man. That really makes me uncomfortable because they think that I am wearing my husband’s boots.” (Female, 31-65 years, Rubavu)*

*“I am a casual worker, so I often go in search for jobs to cultivate for others. The day I wore these boots, and I went to search for work, every person I would meet and ask them for farming work would say ‘aah, someone as rich as you? How can I give a job to an agronomist?’” (Female, 18-65 years, Nyagatare)*

Women also reported that men’s initial reactions to them wearing boots were sometimes negative. Because men viewed boots as footwear meant only for men, they felt that women were overstepping gender boundaries. Although some women reported that these perceptions shifted as men became more accustomed to seeing women wearing boots, not all women experienced this change in attitude.

*“When I started wearing them, he [partner] first seemed okay with the boots, said that they look good. But, after some time he said that women [wearing the boots] were trying to walk over the men.” (Female, 18-65 years, Gisagara)*

*“The first week, when I wore the boots, they [men] laughed at me. But I ignored them. Eventually, I would pass by a group of men, and they would say ‘boots really came on time!’ This is the importance of these boots.” (Female, 31- 65 years, Rubavu)*

Some women explained that community expectations of what they should wear also contributed to increased feelings of intimidation and initially made it harder to wear boots regularly. Even though they had not directly experienced any bullying, their own perceptions about what would be considered acceptable heightened their reluctance to wear boots. This sentiment was mostly reported by older women, who felt that being seen wearing boots would lead to judgement because it conflicted with dominant cultural norms and community practices.

*“When I first received these boots, I took some time to think. I thought about how I would be perceived; an old woman such as myself, wearing boots, kitenge wraps and long skirts, they will laugh at me. I felt intimidated.” (Female, 31-65 years, Gisagara)*

*“The women in my neighborhood questioned if I would be able to walk in the boots. So, I would wear them and try to leave the house. But before I even get to the gate, I would run back in the house, take them off, and put them away … At that time, I was the only one [woman] in my community with the boots, so I feared that they would perceive me as arrogant. So, at first, I was intimidated, I did not want them to think that I act as though I am better than everyone else.” (Female, 31-65 years, Gisagara)*

Participants acknowledged that even though their mindsets had changed, cultural expectations and practices still made it difficult for women to wear boots in public. Therefore, being mocked and ridiculed discouraged most women from wearing boots in places where other people might see them, including markets and village centers. However, even though female farmers still faced stereotypes, all of them reported that this did not deter them from wearing the boots while farming, because they understood the importance of being protected.

*“When we first received these shoes, we went home wearing them. Wherever we passed, men would say that boots are meant for men, that women should not wear them.” (Female, 18-30 years, Gisagara)*

*“The reason we fear wearing these boots outside the farm, such as in the market, is because it has not yet become our culture. We feel that if we do that, people will see us as arrogant, just because we wear boots.” (Female, 31-65 years, Nyagatare)*

Despite persistent cultural norms, participants noticed slow shifts in perceptions and practices, which made it easier for more women to buy boots. The motivation to keep wearing the boots, despite experiencing backlash, stemmed from the support received from their community, local leaders, and partners. Many female farmers expressed that their husbands had been supportive and excited to see them wearing the boots. Some also reported that other men would ask them where to buy the boots, so that they can also purchase a pair for their wives.

*“These boots have motivated other women; they now take money and go to the market to purchase a pair. Things are now totally different, they are changing. In previous times, rural perceptions would make things harder. Many women in this region are now wearing boots” (Male, 18-65 years, Rubavu)*

*“When we first received the boots… we had a Tuesday meeting in the village. So, I went there wearing them. The district executive secretary was at the meeting. When I passed by her, she said that she had seen other women wearing similar shoes and asked where they came from … I told them that I had received them from the health center. She was happy and said that it was a sign of development. She was so happy that, while in the meeting, she mentioned the boots more than five times!” (Female, 31-65 years, Musanze)*

*“They [husbands] were so happy. When I reached home while wearing boots for the first time, my husband asked me, “How did you get these boots?” Other men also were astonished and said, “My God! Could you please direct us where you bought these boots, so that we buy a pair for our wives” (Female, 31-65 years, Rubavu)*

#### Theme 3: Female farmers felt happy, empowered, and proud to wear farming boots.

Across all FGDs, female and male farmers expressed that giving farming boots to women has fostered feelings of confidence, joy, and empowerment. Moreover, women noted that having access to protective footwear made them feel valued and helped restore their sense of dignity. They further reported that the boots often sparked curiosity and admiration from peers and community members, which, in turn, made them feel proud to own and wear them.

*“In my entire life, I had never worn or thought of wearing boots before this. Even when I would see a woman wearing them, I used to laugh at her. I thought she would have walking difficulties. But, on the contrary, when I started wearing them, I would flatter myself and felt proud. I felt like I was walking on air, like God had restored my dignity!” (Female, 18-30 years, Gisagara)*

*“[There is] something interesting about these boots. Women now seem confident and fearless. When they are working at home or in the farms, they no longer fear stepping on sharp objects or stepping on dangerous insects, they just seem very happy and confident.” (Male, 18-65 years, Rubavu)*

*“When I wore these boots for the first time, people stared. When I wore them again, they stared … They started [complimenting me] that I was the most precious/important person in the village because of these boots. And now, I love these boots … we have become stars in the village!” (Female, 31-65 years, Musanze)*

Women frequently praised the design of the boots, remarking that they were unlike other shoes sold in local markets and were distinct from the boots commonly worn by men. They believed that this made the boots well-suited for women and recommended that all women, especially those living with podoconiosis, wear them.

*“Other [women] said that these boots are nice because they are different from men’s boots. These boots you gave us are amazing! They have created a difference between men’s and women’s boots. Women’s boots have laces, but the men’s do not.” (Female, 31-65 years, Rubavu)*

*“When men saw these boots, they seemed excited about them and how they are made. They said that the women’s needs were also considered in the design of the boots, because they have incorporated socks on the insides, to protect the feet.” (Female, 18-65 years, Musanze)*

*“… priority should be given to women living with podoconiosis because these boots can cure it, they can reduce swelling.” (Female, 18-30 years, Gisagara)*

When asked whether the boots matched their desired aesthetics, most women reported no issues. A few respondents mentioned that wearing kitenge wraps and boots was physically uncomfortable, especially while farming, as it often impacted movement. To adapt, some women resorted to wearing trousers while cultivating. Although most women had no issues with wearing trousers, a few stated that they could never wear them and had learned to pair skirts and kitenge wraps with their boots.

*“Experience is everything! I have no problem with wearing a skirt and boots. The more you do it, the more you get used to it, so you can go and cultivate without facing any issues.” (Female, 18-65 years, Musanze)*

*“Wearing a dress with boots is uncomfortable for me, it doesn’t look good. But when I wear trousers, I can insert them in the boots and then tighten them. I feel that it looks better that way. But walking in boots while wearing a dress or a skirt, that is uncomfortable.” (Female, 31-65 years, Nyagatare)*

*“They [women] are now used to wearing trousers underneath the skirts. When they get to the farm, they then remove the skirts and start working. They put the skirts back on when they are done working.” (Male, 18-65 years, Rubavu)*

#### Theme 4: Some farmers who wore the farming boots were labeled as diseased by their communities.

Within male and female FGDs, some farmers who had worn the boots in public said that they were called diseased or disabled, and those with known podoconiosis were ridiculed. Many participants said that this labeling was because the boots were different from what communities were used to, and because they had been given to both healthy individuals and those who suffer from podoconiosis.

*“They kept saying that we are wearing shoes made for disabled people, that these shoes are for people who suffer from podoconiosis.” (Female, 18-30 years, Gisagara)*

*“They told me, ‘Ah, nyirabidido [a derogatory nickname implying a woman with podoconiosis or ugly feet] now has shoes to fit that disease of hers.’ Men would say ‘You see, she is now able to hide those swollen feet, she now has boots to hide them.’” (Female, 18-65 years, Nyagatare)*

Male farmers also reported being made fun of and being discouraged from wearing the boots because their communities viewed the boots’ design as less appropriate for men. Their peers would comment that they are wearing shoes made for children and women. In some instances, these respondents expressed feeling embarrassed or ashamed to wear the boots outside the farm. While male participants reported being teased, none reported ever deciding to farm barefoot because of this mockery.

*“After we received the shoes, we went home wearing them. Some of us, when we ran into other men, they started questioning us. They said that we are wearing kid’s shoes, because of the boots’ height. They kept saying that these shoes are for children, not for men.” (Male, 18-65 years, Gisagara)*

*“What really made them make fun of us, about these boots, are some features like the laces, the insole and the outer cloth. When you meet someone while wearing these boots, they start making fun of you that you are wearing boots that are made of clothes. And when you are with others in town and start making fun of you, you decide never to return to town wearing these boots and go there in a different pair of shoes.” (Male, 18-65 years, Rubavu)*

Further, distributing boots to a subset of the population made recipients stand out. They said that their neighbors questioned why they were the only ones to get boots, which usually led to negative comments about the shoes or the individual wearing them.

*“People will always have something negative to say. There are times that you encounter someone [while wearing the boots], and you hear them say ‘the government knows you, but it doesn’t know us.’ You hear such comments.” (Female, 18-65 years, Nyagatare)*

Even though they were called names within their villages, participants maintained that these community attitudes stem from differing mindsets. Therefore, they felt that this labeling did not define them or discourage them from wearing the boots. Rather than being offended, some participants would use the interactions as an opportunity to raise awareness about podoconiosis and the importance of protective footwear in its prevention.

*“They used to say that people who wear these shoes are people who suffer from podoconiosis. So, we would tell them, ‘Our feet became this way because of walking barefoot, that we are wearing these boots for prevention, so that those who are not suffering from the disease don’t get sick. That’s why we have been given these boots.’ Then they would question why they did not get them. So, we would tell them that the research is still ongoing to see how effective the boots are, that maybe if they are lucky, they will also get them.” (Female, 31-65 years, Musanze)*

*“What they say about us is because of their mindsets and how they perceive things, but we don’t receive it as the truth. We have been educated; we know what these boots are used for. So, when they keep saying that the boots are for children, we know they want to discourage us but that won’t work! We know what we are doing, we have been sensitized, we know how beneficial these shoes are.” (Male, 18-65 years, Gisagara)*

## Discussion

Access to protective footwear is a critical element of global podoconiosis prevention strategies [14,17]. This intervention to test women-centered footwear among rural farmers found that community education and access to farming boots transformed shoe-wearing behaviors and encouraged farmers to challenge prevailing expectations, despite persistent cultural norms and negative labeling. These findings offer vital insights that can inform Rwanda’s 2030 NTD strategy, which aims to eliminate podoconiosis through scaling up integrated clinical services and strengthening locally driven solutions [19,31].

This study demonstrated that combining education with boot distribution encouraged consistent footwear use in rural farmers and was associated with perceived reductions in symptoms (swelling, itching, etc.) among those with podoconiosis. This was similar to NTD endemic communities in western Uganda, where pairing footwear distribution with culturally tailored education increased shoe-wearing by 25% [32]. Further, more than 77% of individuals with podoconiosis in Ethiopia reported reduced swelling after wearing footwear consistently [33]. In Rwanda, podoconiosis prevention programs focus on community sensitization of shoe-wearing [19]. However, simply knowing the benefits of wearing shoes is insufficient to promote consistent use. Research from rural contexts has shown that shoe-wearing is rarely attributed to disease prevention; rather, such decisions are motivated by social expectations and cultural contexts [34]. Moreover, high costs, poverty, culture and tradition, and limited availability remain major barriers to footwear access in Rwanda and elsewhere in sub-Saharan Africa [5,21,22,32,34]. Experiences from Ethiopia have demonstrated the importance of integrated approaches that combine affordable footwear design with strengthening local shoe-production and distribution programs for individuals living with podoconiosis [15,35]. Therefore, podoconiosis programs in endemic regions such as Rwanda should integrate community education initiatives with strategies that address financial barriers, improve the availability of quality shoes in local markets, and tackle other sociocultural dimensions shaping footwear use.

Women in this study associated wearing boots with feelings of empowerment and confidence, despite pervasive and negative gender norms surrounding acceptable attire. This was largely because they felt that the boots were tailored to their needs. These findings support theories that engaging women and integrating their perspectives into NTD prevention programs can promote individual behavior change [36]. In other sectors, including agriculture, participatory approaches that involved women in intervention design processes have similarly reported higher acceptance and adoption of innovations [37,38]. This is because gender transformative approaches that center women’s lived experiences in disease management may help challenge power imbalances, enhance autonomy, and support collective transformation [36,39,40]. In this study, boots appeared to represent more than a prevention tool for some women. They were a pathway through which participants began to challenge social norms and practices that have historically constrained women’s sense of belonging and participation in the agricultural workforce, as well as their engagement in podoconiosis prevention and management. At the same time, however, women reported that persistent stigma and restrictive gender norms continued to shape their experiences and willingness to wear the boots in community settings, with one participant recounting a loss of employment opportunities. These findings suggest that while participatory and gender transformative approaches may foster empowerment and challenge existing norms, such changes are likely to be gradual and require broader efforts to address the structural and economic barriers that limit women’s participation in podoconiosis prevention and management programs. Therefore, to effectively address the needs of vulnerable and underserved communities, podoconiosis and NTD programs in Rwanda and beyond should consider prioritizing human-centered projects that intentionally involve local communities in intervention design and delivery [41,42]. Further, facilitating diverse and interdisciplinary partnerships is essential to promote NTD programs that position women as active agents in disease prevention and control [36].

Praise from local leadership and positive reactions from community members encouraged some women to wear boots in public. Conversely, women who felt that they were the only ones with the boots and those who faced disapproval from their partners or community expressed more reluctance to wear them. These results reemphasize that the effectiveness of NTD programs is shaped not only by innovations, but also the political and social environment in which interventions are implemented [43]. Across sub-Saharan Africa, community leaders and family members play a pivotal role in shaping health behaviors and influencing intervention uptake in podoconiosis programs [44]. Research from other similar contexts shows that involving local leaders and communities in disseminating health information and mobilizing participation in NTD programs can improve intervention acceptability and reshape community attitudes [45–48]. This creates environments that facilitate the adoption of new behaviors, reduce stigma, address power imbalances, and encourage social change. In this study, one government official’s endorsement of women wearing boots as a “sign of development” not only validated female farmers but shaped their intentions to wear boots in public and fostered positive community attitudes towards women’s use of farming boots. These findings demonstrate that the adoption, success, and ownership of NTD interventions are closely tied to their ability to build social legitimacy. Therefore, progress towards meeting Rwanda’s podoconiosis and NTD elimination targets will require ongoing and new programs to prioritize the engagement of local influencers (peers, political and religious leaders, community health workers, etc.) in community sensitization programs, to promote the adoption and acceptance of new behaviors.

Farmers who wore the boots were sometimes labeled as “disabled” or “diseased” by their communities. This occurred because some boots had been distributed to a subset of the community (i.e., individuals living with podoconiosis) and because the boot design was uncommon in local markets. This labeling reflects broader processes of stigma, whereby differences that are identified and labeled by a dominant group can lead to negative stereotyping, social exclusion, discrimination, and status loss [49]. Similar stigmatization of custom-made shoes in podoconiosis management was documented in Ethiopia, where individuals with podoconiosis who received shoes from the Mossy Foot program reported discriminatory experiences that prompted them to stop wearing the shoes or to restrict their use to special occasions [20]. Contrary to their findings, this study’s participants confronted this labeling directly, instead of giving up on their boots. Instances of mockery and enacted stigma became opportunities to raise awareness about podoconiosis, and the importance of consistent footwear use in its prevention. Negative reactions reported in both studies reflect broader community misunderstandings about podoconiosis, which is often poorly understood and believed to be contagious [5]. By resisting stigma to educate their communities, farmers in this study used knowledge gained from the education campaigns to challenge misconceptions and reframe the footwear from a symbol of illness to one of protection and prevention. This aligns with broader literature highlighting community education as an important strategy for engaging affected communities as partners in addressing misinformation and stigma associated with podoconiosis [50–52]. However, findings also suggest that footwear interventions may unintentionally reinforce disease-related labels, particularly when initiatives target only a subset of the population or when designs visibly distinguish recipients from other community members. Therefore, future scale-up efforts should pair footwear distribution with community-wide sensitization and implement distribution approaches that avoid singling out specific groups. Involving entire communities in such initiatives may strengthen the acceptability of footwear-focused podoconiosis programs.

A few limitations should be considered when interpreting the findings of this study. The period to test the footwear prototype (one month) was relatively short, so the results might not capture long-term changes in shoe-wearing behaviors and experiences. The study did not test the boots’ effectiveness in managing podoconiosis and, therefore, cannot make any conclusions about the prototype’s ability to reduce or stop disease progression. The research was conducted in rural and endemic communities with the highest podoconiosis prevalence in Rwanda, providing distinct perspectives that might not apply to urban and low-prevalence settings. Farmers were aware that they were participating in a research study and would be later asked to evaluate the footwear prototype, which may have temporarily influenced their shoe-wearing behaviors (Hawthorne effect). In addition, the boots were provided free of charge, so perceptions about the prototype’s acceptability and use may differ in contexts in which farmers would have to purchase the boots themselves. Because data were collected through group discussions, farmers may have potentially expressed experiences and opinions that they might deem favorable or socially acceptable. Lastly, the gender and behavioral concepts explored in the study are uniquely shaped by the cultural and social beliefs, practices, and contexts of the communities in which the intervention took place, which might not reflect the lived experiences of other communities in Rwanda and beyond. Despite these limitations, this study offers valuable insights into the acceptability and uptake of a custom-made farming boot in podoconiosis endemic communities, which could inform future research and interventions in Rwanda. Further, it highlights the importance of combined academia-industry-community partnerships in implementing innovative and community-centered solutions to the challenges faced by impoverished and marginalized populations.

## Conclusion

This community-based project demonstrated how testing a women-centered farming boot co-designed with rural farmers promoted consistent footwear use and fostered feelings of empowerment among women who wore the boots, despite facing discouraging community reactions and restrictive gender norms. Our results highlight the value of contextualized interventions in promoting the acceptability and ownership of locally driven solutions and in addressing the cultural, gender and social dynamics of podoconiosis management in impoverished communities. Achieving Rwanda’s podoconiosis elimination targets will require, among other efforts, prioritizing gender transformative interventions that address social and cultural barriers to footwear use, promote equitable access to farming boots, and enable affected communities to contribute to disease control efforts. On a broader scale, our findings highlight the importance of implementing innovative podoconiosis and NTD elimination strategies that are socially and culturally grounded in the lived experiences of at-risk communities. Overall, the continued success of podoconiosis elimination programs in Rwanda will require recognizing equitable access to protective footwear as a public health priority.

## Supporting information

S1 Text English focus group discussion guide. (DOCX)
