# Supplementary material for: “My feet are no longer swollen”: Testing a co-designed podoconiosis protective footwear among rural female farmers in Rwanda
Source: PLOS Glob Public Health. 2026 Oct 6;6(10):e0007114. doi: 10.1371/journal.pgph.0007114 (PMC13641362; doi:10.1371/journal.pgph.0007114)
Supplement: Supplementary file 1 [file pgph.0007114.s001.docx]

# **Prototype Testing English Focus Group Discussion Guide**

Moderator:

Notetaker:

District:

Sector:

Date:

Time started/ended:

Number of participants:

*[Facilitator script]*

Hello, welcome back, we are happy to see you again. My name is [________] and this is my colleague [__________]. In a moment we will all introduce each other again.

Our goal today is to hear your perspectives about the shoes that you tried over the last month. Your opinion and advice will be very important to the success of this project. Please share your thoughts honestly.

***[Remind participants about the consent forms that they signed during the first FGD and that those rights still apply.]***

Similar to our last meeting, let us adhere to basic ground rules to ensure that everyone feels comfortable to speak.

- Only one person speaks at a time
- There is no order on who speaks first, anyone who has something to share is welcome to start speaking
- Kindly avoid interrupting each other
- Please respect participants who are sharing opinions that differ from your own

Does anyone have any questions they would like to ask before we begin?

***[Assign participants a number from 1 to 10]***

Okay, I will turn on the recorder and we can begin our conversation.

***[TURN ON THE RECORDER]***

Let’s go around and introduce ourselves. State your given discussion number, your age, how long you have been a farmer for, the type of farming that you do, and what you like most about your community.

***[As participants are speaking, please fill in the following table]***

| FGD participant given ID | Age | Sex | Years practicing farming | Type of farming (livestock, crops, both) |
| --- | --- | --- | --- | --- |
| *E.g. 9* | *23* | *F* | *2* | *Animals (goats and sheep)* |
|  |  |  |  |  |
|  |  |  |  |  |
|  |  |  |  |  |
|  |  |  |  |  |
|  |  |  |  |  |
|  |  |  |  |  |
|  |  |  |  |  |

***[Use the following questions to guide the discussion. Allow time for participants to respond to each other’s comments and encourage all participants to speak. Ask follow-up questions when an answer is unclear.]***

1. To start, we would like each person to share their experiences testing the shoes. What did you like? What did you dislike?

*[Facilitator should fill the following flip chart]*

| **Likes** | **Dislikes** |
| --- | --- |
|  |  |

*[Facilitator will then guide a ranking and piling exercise to understand the top 3 likes and dislikes. Each participant receives 10 stones and ‘votes’ for the factors that most strongly influenced their overall impression. Participants should explain why they are voting a particular way]*

2. Let’s consider your top 3 dislikes. What could be done to solve them? Does anyone disagree?

a. Dislike #1

b. Dislike #2

c. Dislike #3

*[Thank participants for their ideas and end this session with a summary of the most popular solutions. State that you will report back these opinions to the design team and remind participants that changes might not necessarily be possible]*

3. In our first meeting we talked about various activities where people wore shoes. Are the shoes you tested suitable for those activities? Why or why not?

*{Probe: Are there other activities where these shoes are useful?]*

| **Activity** | **Yes** | **No** | **Maybe** |
| --- | --- | --- | --- |
| Household chores |  |  |  |
| Farming |  |  |  |
| Cutting grass |  |  |  |
| Looking after livestock |  |  |  |
| Fetching water |  |  |  |
| Going to the market |  |  |  |
| Attending social activities (e.g., church) |  |  |  |

4. Let’s take another look at the TPP that you developed during our first meeting.

*[Facilitator should prepare this flip chart in advance with all TPP criteria from the Objective 1 FGDs. As participants speak, populate the Yes/No/Maybe columns with checkmarks. Probe their votes to understand the reasons]*

| **TPP criterion** | **Yes** | **No** | **Maybe** |
| --- | --- | --- | --- |
|  |  |  |  |
|  |  |  |  |
|  |  |  |  |
|  |  |  |  |
|  |  |  |  |

5. If one of your female family members needed footwear, would you recommend these? What about a male family member?

6. What do you think these shoes should cost at the market? As they are now, would you buy them? Why or why not?

7. During the last month, did the amount of time you spent wearing shoes change? Did it increase or decrease? Why or why not?

****End of Discussion: Thank the participants for joining the discussion. Ask them if they have any questions or anything they would like to share with the team before concluding the FGD.***
